# Exposure of trophoblast cells to fine particulate matter air pollution leads to growth inhibition, inflammation and ER stress

**DOI:** 10.1371/journal.pone.0218799

**Published:** 2019-07-18

**Authors:** Mary Familari, Åsa Nääv, Lena Erlandsson, Robb U. de Iongh, Christina Isaxon, Bo Strandberg, Thomas Lundh, Stefan R. Hansson, Ebba Malmqvist

**Affiliations:** 1 School of BioSciences, University of Melbourne, Parkville, Australia; 2 Lund University, Faculty of Medicine, Department of Clinical Sciences, Division of Obstetrics and Gynecology, Lund, Sweden; 3 Department of Anatomy and Neuroscience, School of Biomedical Sciences, University of Melbourne, Parkville, Australia; 4 Department of Ergonomics and Aerosol Technology, Lund University, Lund, Sweden; 5 Division of Occupational and Environmental Medicine, Lund University, Lund, Sweden; 6 Lund University, Skåne University Hospital, Department of Clinical Sciences Lund, Division of Obstetrics and Gynecology, Lund, Sweden; Chinese Academy of Sciences, CHINA

## Abstract

Ambient air pollution is considered a major environmental health threat to pregnant women. Our previous work has shown an association between exposure to airborne particulate matter (PM) and an increased risk of developing pre-eclamspia. It is now recognized that many pregnancy complications are due to underlying placental dysfunction, and this tissue plays a pivotal role in pre-eclamspia. Recent studies have shown that PM can enter the circulation and reach the human placenta but the effects of PM on human placental function are still largely unknown. In this work we investigated the effects of airborne PM on trophoblast cells. Human, first trimester trophoblast cells (HTR-8/SV) were exposed to urban pollution particles (Malmö PM2.5; Prague PM10) for up to seven days *in vitro* and were analysed for uptake, levels of hCGβ and IL-6 secretion and proteomic analysis. HTR-8/SVneo cells rapidly endocytose PM within 30 min of exposure and particles accumulate in the cell in perinuclear vesicles. High doses of Prague and Malmö PM (500–5000 ng/ml) significantly decreased hCGβ secretion and increased IL-6 secretion after 48 h exposure. Exposure to PM (50 ng/ml) for 48h or seven days led to reduced cellular growth and altered protein expression. The differentially expressed proteins are involved in networks that regulate cellular processes such as inflammation, endoplasmic reticulum stress, cellular survival and molecular transport pathways. Our studies suggest that trophoblast cells exposed to low levels of urban PM respond with reduced growth, oxidative stress, inflammation and endoplasmic reticulum stress after taking up the particles by endocytosis. Many of the dysfunctional cellular processes ascribed to the differentially expressed proteins in this study, are similar to those described in PE, suggesting that low levels of urban PM may disrupt cellular processes in trophoblast cells. Many of the differentially expressed proteins identified in this study are involved in inflammation and may be potential biomarkers for PE.

## Introduction

Ambient air pollution, considered a major environmental health threat to pregnant women and their offspring [1], is a mixture of gases and particles in the air. When present in sufficient concentration, or of sufficient hazardous types, it affects human health (1). One main source of airborne particulate matter (PM) in urban regions are motor vehicle emissions, which can also contain various metals and toxic compounds such as mutagenic polyaromatic hydrocarbons (PAH) [2].

Previous epidemiological studies by us [3, 4] and others [5–8] have reported the adverse effects of ambient air pollution on pregnancy-related maternal health as well as affecting the health of the offspring by complications such as low birth weight and pre-term births. In particular, we have shown that the severity of disease is linked to increased exposure to traffic-related air pollution [3, 4, 9]. Dadvand and colleagues have also found that air pollution from traffic-related sources has the most impact for adverse pregnancy outcomes [10]. The risk estimates for air pollution are comparable to those for high maternal age and being overweight [3].

Air pollution levels in urban cities of Sweden generally meet the WHO guidelines for annual mean concentrations of different fractions of PM [11]. However, we have reported an association between air pollution exposure during pregnancy and a higher risk of pre-eclampsia (PE) for some Swedish cities [3, 4], suggesting that even low levels of air pollution are hazardous.

PE is a clinical syndrome afflicting 3–7% of pregnant women, and is one of the leading causes of maternal mortality and morbidity, especially in developing countries [12]. With its aetiology still largely unknown, the diagnosis of PE is based on maternal clinical manifestations; high blood pressure and proteinuria manifesting after 20 weeks of gestation. The predominant view suggests that the disease evolves in the utero-placental unit, most likely due to defective placentation leading to uneven blood perfusion, inflammation and oxidative stress [13], and as it progresses, gives rise to maternal endothelial damage causing the clinical manifestations. Placental oxidative stress also triggers immune and inflammatory responses in the mother [14]. Although normal pregnancy is characterised by a mild systemic inflammatory response, in PE these responses are more extensive [15]. However, the role of inflammation in PE alone, or with intra-uterine growth restriction, is not well understood. One suggested pathway between air pollution and pre-eclampsia (PE) is through the placenta [9].

It is now recognized that many pregnancy complications are due to underlying placental pathology or dysfunction [16]. In addition, we [3, 4] and a number of others [5–8, 17–21] have confirmed a link between levels of PM pollution and pregnancy complications such as preterm births and pre-eclampsia, disorders in which placental dysfunction plays a pivotal role. It is well-established in rodents that inhaled PM during pregnancy not only reach the placenta but affect this tissue and the fetus [22, 23]. In particular, PM cause increased levels of cytokine release into the circulation, and inflammation within the placenta [24–26].

In humans, Nemmar and colleagues [27] demonstrated that inhaled labelled carbon particles (less than 100nm) could be detected in the circulation of healthy volunteers within 1 minute following exposure and could still be detected 60 minutes later. In addition, an increase in the release of proinflammatory cytokines (e.g. IL-6, IL-1b) into the human circulation following exposure to PM has been demonstrated [17]. Changes in placental functions including altered gene expression and increases in markers of oxidative stress [28, 29], mitochondrial dysfunction and altered methylation profiles [30–32], and endothelial and villi pathology [21] have been reported following inhalation of PM during human pregnancy. More recently, Qin and colleagues found that exposure to high levels of urban PM2.5 (particles ≤ 2.5 μm) induced cell-cycle arrest, inhibited migration and invasion of extravillous trophoblast cells [33]. A recent report by Liu et al. [34] indicated that carbonaceous PM are able to translocate from the lungs to the placenta and are engulfed by resident macrophages (Hofbauer cells).

In this study we have examined the effects of urban PM2.5 and PM10 (particles ≤10μm) on cultured first trimester trophoblast cell endocytosis, cell growth and gene expression.

## Materials and methods

### Urban particulate matter (PM)

Two sources of urban PM were used: Malmö PM2.5 (≤ 2.5 μm) and Prague PM10 (average size < 4 μm). Prague PM10, a standard reference material, was obtained from Sigma Aldrich (NIST2786). Originally collected in 2005 in Prague, Czech Republic, PM10 is representative of PM collected in an urban environment and not specific for any area of Prague. Prague PM10 has been chemically characterised and was used to compare the Malmö PM chemical composition analysis undertaken in this study, and to test their biological activity on human trophoblast cells. Apoptosis and increased levels of reactive oxygen species following exposure of human bronchial epithelial cells to Prague PM10 (up to 500 μg/ml) has been reported [35].

Malmö PM2.5 particles were collected over 26 days in April-May 2017 at 3 m height, and 4 m from a street crossing with an annual average daily traffic (2017) of 28000 vehicles in central Malmö, using a high-volume cascade impactor (BGI900, Mesa Labs, USA). The impactor samples air (0.9 m^3^/min) and collects all particles smaller than 2.5 μm on a polypropylene filter. The collected PM2.5 consists of particles generated by traffic and biomass burning, from both within the city and from east/south east (Poland) and west (Copenhagen). Time resolved mass concentration measurements of PM2.5 were conducted with a tapered element oscillating microbalance (TEOM 1400AB) and of black carbon (soot) and organic carbon content with an aethalometer (AE33). These analyses indicated mean PM2.5 was 6.1 ± 3.1 μg/m^3^ and 23.9 ± 10.3% of the particles were generated by biomass burning. Additional sources are mainly traffic-related, both locally generated and in-transported particles. The collected particles were extracted using pure methanol and dried in a vacuum evaporator. The dried collected particles were analysed by gas chromatography-mass spectrometry (GC-MS) for PAH [36] and by inductively coupled plasma mass spectrometry (ICP-MS) for metal composition according to standard procedures. While the overall composition of the Prague and Malmö PM sample were similar, the levels of PAH in the Malmö PM sample were 1.5–5-fold lower than in the Prague PM sample (NIST Certificate of Analysis Standard Reference Material 2786, 2016, Gaithersburg, MD, USA), depending on the particular PAH. Notably, greater concentrations (2–100-fold) of metals were found in the Prague PM samples than in the Malmö PM sample (See Supporting information S1 Table). Analysis of nitro-PAHs and oxy-PAHs were not included in this study.

Both PM samples were provided as powdered material that required sonication for resuspension, which is standard procedure in air pollution studies using cultured cells [35, 37, 38]. To disperse PM in solution, samples were subjected to a two-step sonication in 0.5 ml of RPMI in 1.5 ml tubes (Eppendorf). Sonication with the probe immersed into the solution, at 50 W, 0.05 cycle, 20% amplitude for 1 min was undertaken using UP50H Ultrasonic Processor, (Hielscher Ultrasound), followed by ultrasonication at 4°C, 120W, 15 min using Mettler Electronics Ultrasonic Bath. To avoid settling of PM, aliquots were sonicated in 500 μl RPMI immediately before use. Unexposed control cells were treated with RPMI without PM, which was subjected to the same sonication procedure. Cells were exposed to PM in RPMI, or RPMI alone (unexposed vehicle controls), or the supernatant of dispersed PM after centrifugation for 10 min at 14,000 rpm at room temperature.

### Placental exposure considerations

While the actual exposure rate of the placenta to PM is not known, it is well established that PM ≤ 2.5 μm, can and do bypass lung epithelial phagocytic cells to enter directly into the circulation [39]. The concentrations of PM used in this study are based on two key factors: 1. Our previous epidemiological studies showing that there is an association between women exposed to ambient air pollution in Malmö and low birth weights and pre-term births [3–4]; 2. The average daily mean ambient PM2.5 of 20–30 μg/m^3^ in Malmö Sweden, (compared to Swedish national average < 10 μg/m^3^). Therefore, we used the following parameters to estimate a realistic exposure dose: daily mean ambient PM 2.5 rate of 25 μg/m^3^ in Malmö Sweden, changes in cardio-respiratory physiology during pregnancy [40] and lung PM clearance capacity [39, 41]. We calculated that pregnant women were exposed to between 50–500 ng of PM2.5 per day, and thus the dose-response curve for our studies ranged from 0.5 to 5000 ng/ml. Details are provided in Supporting Information S1 Text.

### Trophoblast cell culture

Primary placental trophoblast cells can be isolated from term placentae. However, the yield of cells is variable, and due to genetic heterogeneity, the levels of secreted factors among placentae are also highly variable. To ensure reproducibility, we used the HTR-8/SVneo cell line, a human first trimester transformed placental trophoblast cell line, purchased from American Type Culture Collection (ATCC Cell Lines, CRL-3271, Lot Number 64275781). Cells were expanded in RPMI culture medium (RPMI-1640 Medium Gibco, ThermoFisher Scientific) supplemented with 5% fetal bovine serum (Life Technologies) and 100 units/ml Penicillin-Streptomycin (Gibco)]. Six-well culture plates (ThermoFisher) were seeded with 3 x 10^5^ (acute treatment, 48 hours) in 1 ml of RPMI, and exposure to urban particulate matter at varying doses from 5 ng to 5000 ng/ml began 24 hours later. Cells were exposed to a single dose of PM without media change for 48hrs. The initial seeding capacity throughout this study was chosen to prevent cells reaching confluence in the exposure period, which may have confounded our results.

### Analysis of trophoblast cell function and gene expression

To investigate whether PM had effects on markers of placental biology, we harvested culture medium for determination of concentrations of secreted hCGβ and progesterone (Clinical Biochemistry Laboratory, Lund Hospital), as well as IL-6 (human IL-6 ELISA Kit, Invitrogen), 48 hours after exposure of PM. All treatments were assayed in triplicate in each experiment. Following removal of culture medium, cells were washed twice, trypsinized, harvested and counted. All experiments were repeated three times and data expressed as mean ± SD and analysed by one-way ANOVA followed by post-hoc tests, incorporating a Bonferroni correction for multiple comparisons, where p < 0.05 was considered significant.

Previous studies have shown altered expression of a range of genes in PE [42, 43]. To investigate whether these genes were altered after PM exposure, we used custom qPCR arrays (see Supporting Information S1 Text for gene expression methodology and selection rationale.) to examine expression of these genes after 48hours of exposure to PM.

### Analysis of endocytosis

HTR-8/SVneo cells were plated on glass coverslips, coated with 1% gelatin (Sigma Aldrich) in phosphate buffered saline (PBS, Gibco) at a density of 1 x 10^3^ cells per coverslip before placing in 12-well plates with 1 ml RPMI. Cells were plated directly onto coverslips (for histological staining), or after pre-staining in 2 ml of 2 x 10^−6^ M PKH67 (green fluorescent cell linker mini kit for general cell membrane labelling, MINI67, Sigma Aldrich) prepared according to manufacturer’s protocol, for 5 min at room temperature.

After 24 hours, cells were exposed to different doses of Malmö or Prague PM in RPMI; control cells were unexposed. Cells received a single dose of PM, and media was changed every 48 hours. The cells were harvested after a further 24–168 hours of PM exposure, washed four times in PBS, before fixing in 4% paraformaldehyde (in PBS, pH 7.0; Sigma Aldrich) for 20 min at room temperature. Following fixation, cells were washed three times in PBS before staining in hematoxylin and eosin (H&E, n = 2 experiments); or washed once in PBS, stained with DAPI nuclear stain for 10 min before a further two washes in PBS (fluorescence studies, n = 4 experiments).

### Image analysis

Bright field images were taken using an Olympus BX60 microscope with cellSens Entry micro-imaging software. Fluorescent images were taken using a Zeiss LSM800 Airyscan Confocal with Zen Blue 2.1 Software at the Biological Optical Microscopy Platform at the University of Melbourne. In each of four experiments, fluorescent images (one per quadrant) of the PKH67-labelled vesicles and DAPI-stained nuclei were taken per sample.

Image J software [44] was used to quantify the number of PKH67-labelled vesicles and DAPI-stained nuclei in the same field. Data are expressed as mean ± SD number of PKH67-labelled vesicles/nuclei or number of nuclei per field and analysed by 2-way ANOVA with post-hoc tests (Holm-Sidak correction) using GraphPad Prism 7.04.

### Quantitative proteomic analysis

For proteomic analysis, 3 x 10^5^ HTR-8/SVneo cells were seeded in T-75 culture flasks (Nunc, ThermoFisher Scientific) and exposed to Prague PM (50 ng/ml) once (n = 3, Acute) or daily (n = 3, Chronic) and cells harvested after 48 hours (single dose without media change) or 7 days (both daily doses and media changes), respectively, and compared to unexposed controls (n = 4) receiving vehicle only with media changes daily for the same periods. Cells were washed 4 times in PBS, before removal with trypsin, pelleted and stored at -80°C for analysis.

Proteomics on cell pellets was performed at the Proteomics Core Facility at Sahlgrenska Academy, University of Gothenburg (http://proteomics.cf.gu.se/proteomics). A Tandem Mass Tag (TMT) set containing ten isobaric tags or labels (10-plex) allowed comparison of ten samples in one experiment for analysis by Mass Spectrometry (MS). In total, 100 μg protein per sample was trypsin digested into peptides and uniquely tagged at the N-terminals before analysis by nano-Liquid Chromatography-MS/MS. Proteins were identified in MS-raw data using Proteome Discoverer version 1.4 (ThermoFisher Scientific) against the Human Swissprot Database version March 2017 (Swiss Institute of Bioinformatics, Switzerland) and relative quantification was based on the ratio of reporter ion intensities/isobaric labelling). The low variability and the high sensitivity of the methodology used at the Proteomics Core Facility, University of Gothenburg, allows fold change of ≥1.2, if p-value is <0.05, to be considered significant. Student’s *t*-test was used to determine significant fold changes compared to unexposed controls, and together with Ingenuity Pathway Analysis (IPA, Qiagen) and Reactome Analysis (https://reactome.org/) to determine the biological relationships. Heatmaps were generated by Morpheus, https://software.broadinstitute.org/morpheus) for differentially expressed proteins (at p < 0.05 level of significance, FC > 1.2), and IPA analysis was used to generate diagrammatic representation of significant pathways and networks affected by PM exposure.

Reverse Phase Protein Arrays (RPPA) were performed at the Victorian Centre for Functional Genomics (VCFG), and the ACRF Translational RPPA platform at Peter MacCallum Cancer Centre Melbourne, for proteomic validation studies as previously described [45]. Briefly, snap frozen protein lysates of PM exposed and control cells (n = 3 samples per group) were homogenised in CLB1 buffer (Zeptosens, Bayer), and quantified. Using a Caliper ALH3000 liquid handling robot (Perkin Elmer), samples were serially diluted before spotting onto ZeptoChips (Zeptosens) in duplicate using a Nano-Plotter-NP2.1 non-contact arrayer (GeSim). Chips were blocked, incubated with pre-validated primary antibodies diluted 1:500 for 20 hours before applying Alexa Fluor647 anti-rabbit secondary antibody (1:1000, 4 hours) (#Z-25308, ThermoFisher Scientific). Zeptosens instrument and software (version 3.1) were used to scan and calculate the relative fluorescence intensity. All samples were normalised to the background values reported in the secondary antibody-only negative controls. The antibodies used in the array, have been experimentally verified and highly-specific for signalling pathway components but include only a limited selection of pathway proteins in phosphorylated and non-phosphorylated form.

## Results

### Pollution particles induce decreased cell growth and increased endocytosis

**Fig 1:**
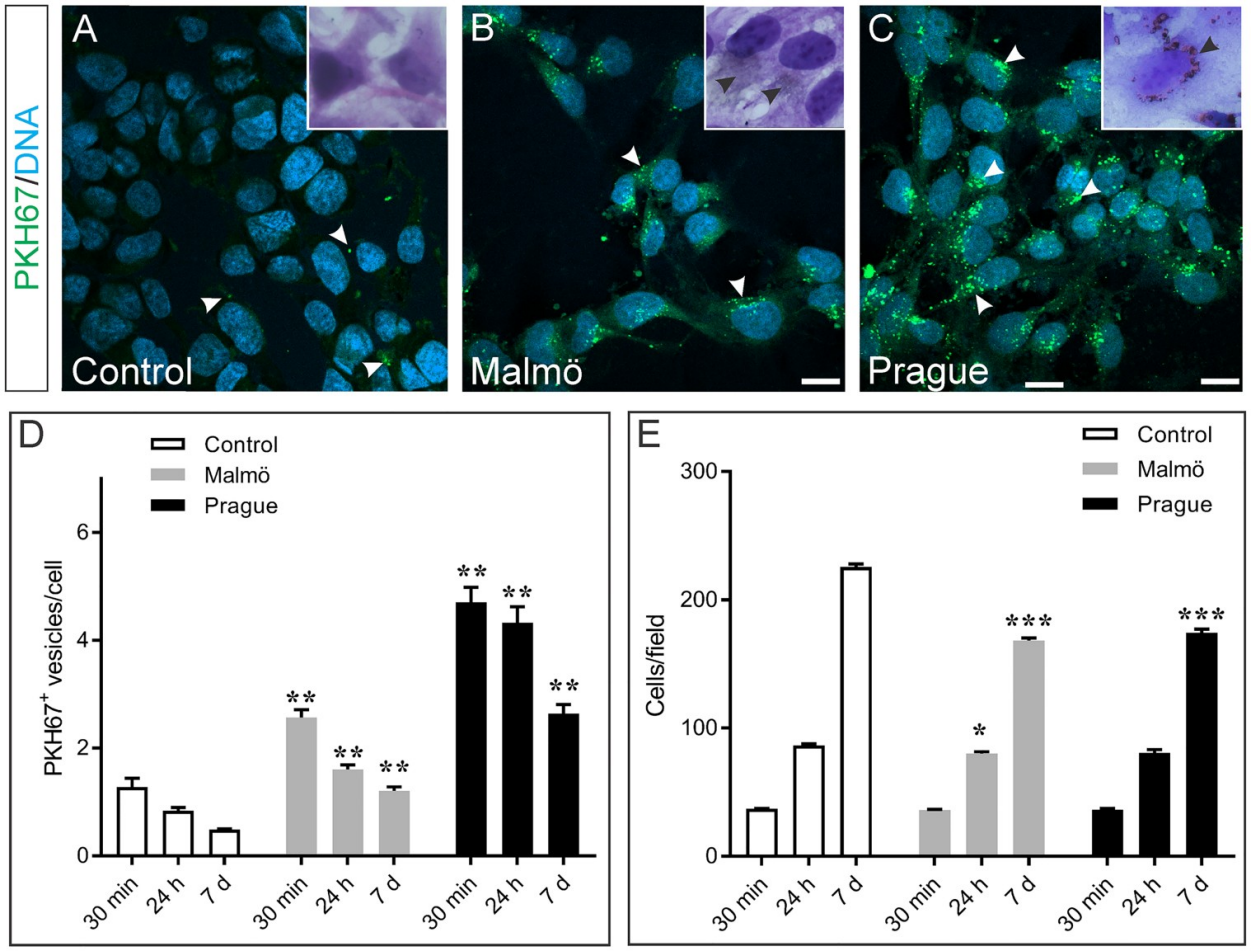
Endocytotic responses of HTR-8/SVneo cells treated with Malmö and Prague PM. **(A-C):** Fluorescence images showing PKH67 membrane stain (green), nuclei DAPI stain (blue) after 24 hr of PM exposure. Control cells show very few PKH67-stained endosomes (**A**, arrowhead). Cells exposed to Malmö PM show many small endosomes IB, arrowheads), whereas larger endosomes were detected in the Prague-treated cells (**C**, arrowheads), often in perinuclear region. Scale bars: 20 μm. (**D-E):** Quantification of numbers of endocytotic vesicles per cell (**D**) and cell number per field (**E**). Histograms display mean ± SEM. Significance of statistical analyses of particle-treated cultures with respective controls are indicated by: *, P<0.05; **, p<0.001; ***p<0.0001 (2-way ANOVA and t-test with Holm-Sidak correction for multiple comparison). See Supporting information S1 Fig and S1 Movie showing uptake of endocytic vesicles.

To examine the effects of the pollution particles on placental cells we exposed HTR-8/SVneo cells to Malmö and Prague particles over a culture period of 7 days (Fig 1) and examined the endocytotic responses, using the PKH67 fluorescent dye, and counted the number of cells at each time-point using DAPI nuclear dye.

PKH7 is a dye that labels cell surface membranes as well as endosomes and phagosomes [30]. There was very little labelling of internalised vesicles by PKH67 in unexposed control cells (Fig 1A). By contrast, in the Malmö PM-treated cells there were numerous small, fluorescently labelled vesicles in the cytoplasm (arrowheads, Fig 1B). However, in the Prague PM-exposed cells these fluorescently labelled vesicles were more numerous and larger (arrowheads, Fig 1C). Similarly, images of H&E stained cells showed fine particles (inset, Fig 1B) in the Malmö PM exposed cells and much larger particles in the Prague PM-exposed cells (inset, Fig 1C).

Quantification of PKH67 fluorescently labelled endocytotic vesicles in control and pollution particle-exposed HTR-8/SVneo cells showed statistically significant, time-dependent decreases in the numbers of endocytotic vesicles per cell from 30 min to 24 h and 7 days in all cultures (Fig 1D), consistent with this being a pulse labelling experiment and that the vesicle-bound fluorescent marker is likely processed during the culture period. However, both Malmö and Prague particle-exposed cells showed a significantly higher number of endocytotic vesicles per cell (p<0.001) when compared to the respective control cultures at each time-point (Fig 1D).

Observation of the cells during the culture period suggested that in the particle-exposed dishes there were fewer cells. Quantification of cell numbers at 30 min, 24h and 7 days after exposure showed that all cultures (vehicle-controls and particle-exposed) showed significant increases (p<0.0001) in cell number at each time-point over the 7-day culture period (Fig 1E), consistent with the normal expansion of these cells in culture. However, at 7 days, both the Malmö and Prague particle-exposed cultures showed significantly reduced numbers of cells (p<0.0001, Fig 1E) compared to controls, suggesting the growth of the cells in particle-exposed cultures was significantly inhibited. At 24 h in culture, only the Malmö cells showed a significant decrease (p = 0.03) in number, compared to respective controls (Fig 1E). There was no significant difference between the two particle-exposed cultures at any time-point (Fig 1E).

### PM effects on protein secretion

**Fig 2:**
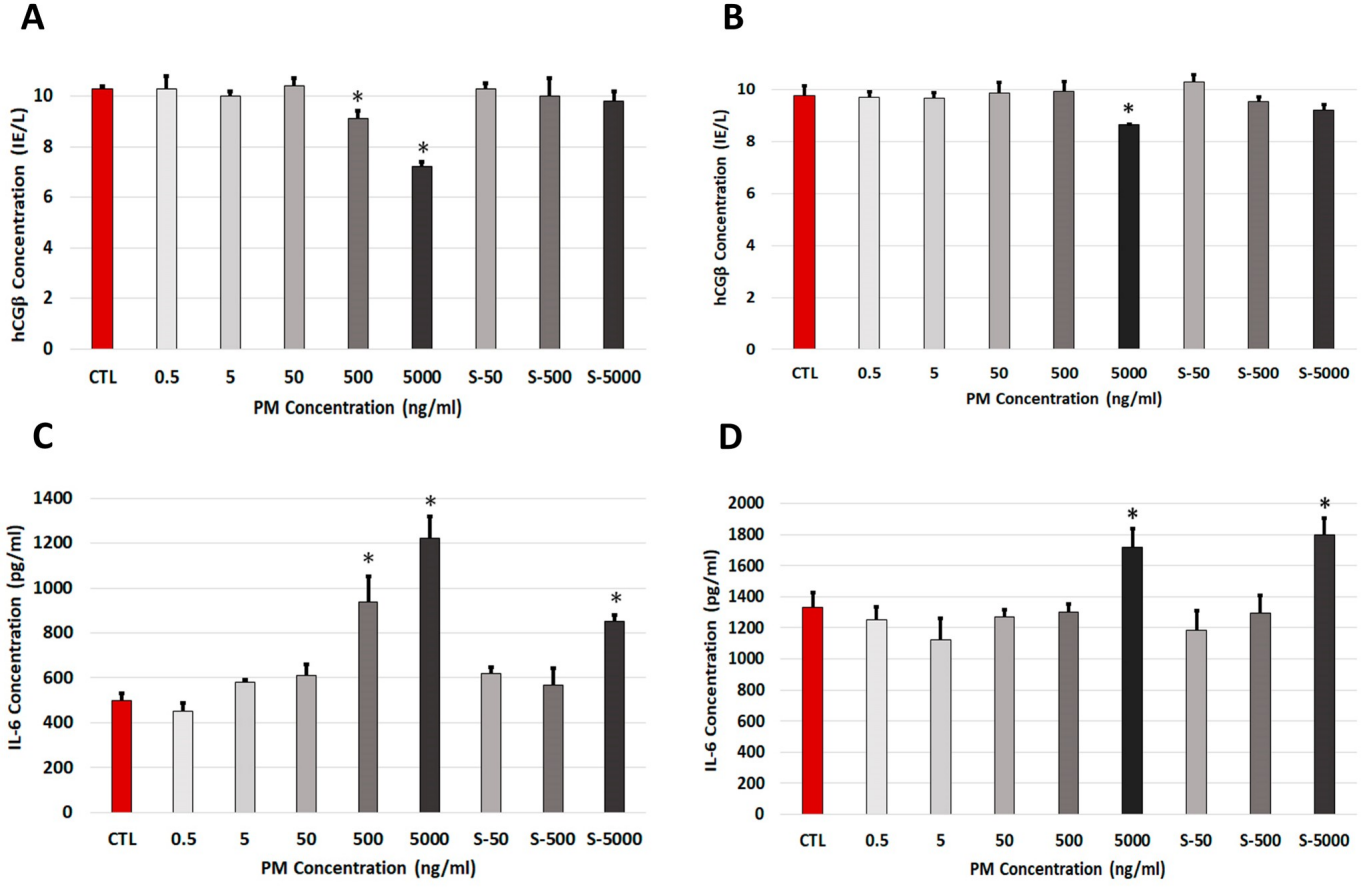
Effect of PM exposure on trophoblast protein secretion. The level of hCGβ (**A, B**) and IL-6 (**C, D**) secretion was measured in culture supernatant following exposure for 48 hours with varying doses of Prague (**A, C**) or Malmö (**B, D**) PM or PM-conditioned media. Results are expressed as ± S.D of triplicate wells. * p < 0.05. Abbreviations: Control (**CTL**), PM-conditioned media (**S-**).

To investigate whether PM had effects on markers of placental biology, we examined the levels of several factors [46]: hCGβ (specific trophoblast pregnancy marker), IL-6 (marker of placental inflammation), progesterone (marker for trophoblast adhesion via MMP9) in the culture media. Exposure of HTR-8/SVneo cells for 48 h to varying concentrations of PM showed that only high doses of Prague (500 and 5000 ng/ml, Fig 2A) and Malmö PM (5000 ng/ml, Fig 2B) significantly decreased hCGβ secretion. Interestingly, exposure to Malmo PM supernatant at 500 or 5000 ng/ml also decreased hCGβ secretion (p < 0.01). Progesterone secretion was unaffected (see Supporting information S2 Fig).

By contrast, PM treatment for 48 h with high concentrations of Prague PM (500 and 5000 ng/ml) and Malmö PM (5000 ng/ml), caused significantly increased secretion of IL-6 (Fig 2C). Similarly, treatment of cells with the culture supernatants (500 ng/ml Prague PM and Malmö PM) also caused increased IL-6 secretion.

### PM effects on gene expression

As exposure to the PM affected cellular secretion, we also examined whether there were changes in gene expression by quantitative RT-PCR. Exposure to Malmö or Prague PM at varying doses from 5 ng to 5000 ng/ml for 48 hours did not affect expression of any of the assayed genes (*GAPDH*, *18S*, *NFE2L2*, *MMP9*, *COX10*, *HMOX1*, *HIF1A*, *PECAM1* and *ITGA5*) compared to the expression in unexposed control cells (See Supporting information S2 Table for gene expression data).

### Proteomic analyses

**Fig 3:**
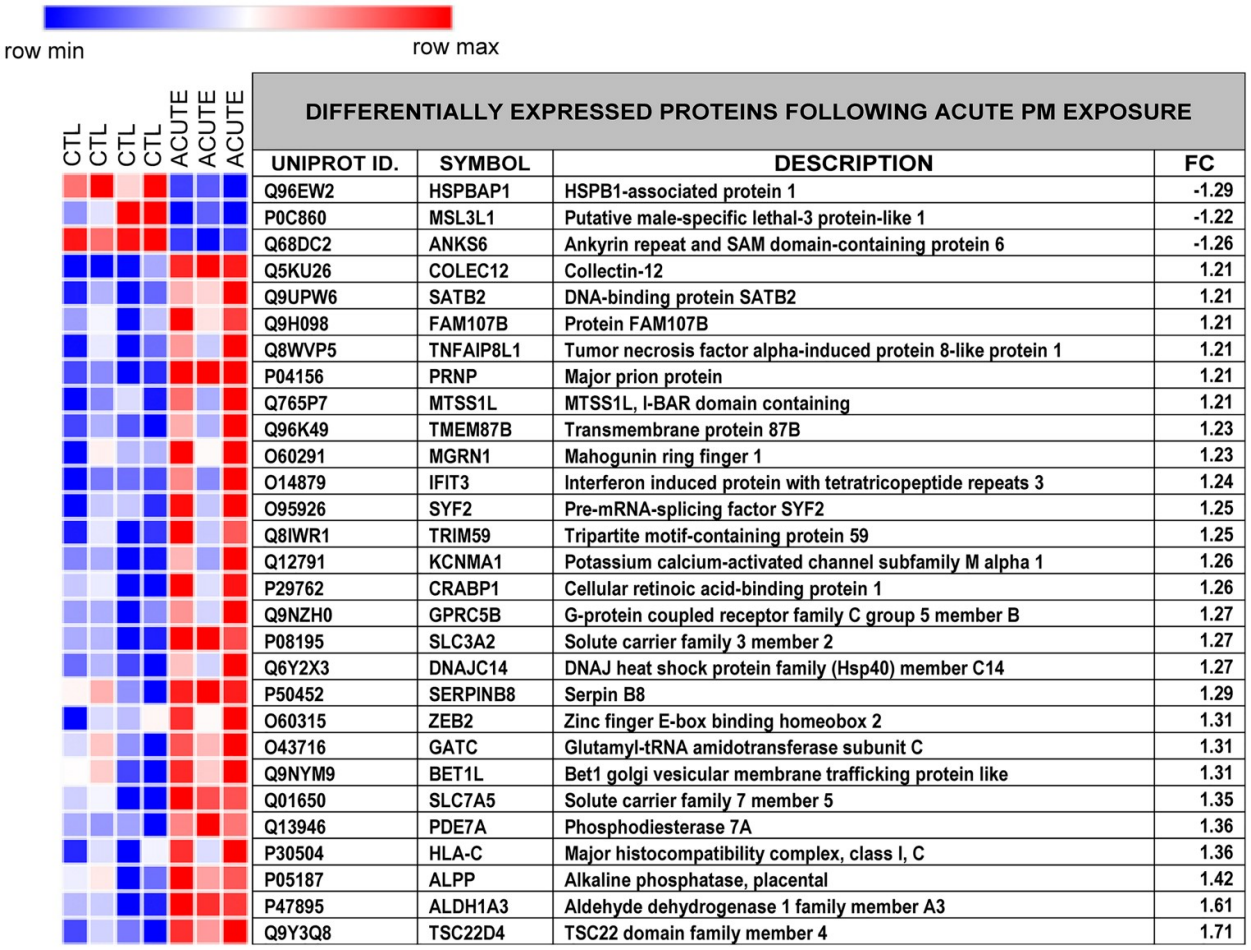
Differentially expressed proteins following acute Prague PM exposure. Heatmap showing replicate expression intensities for control (CTL) and acute exposed samples with matching table showing levels of differential protein (UniProt code and description) expression (FC, fold change) following acute PM exposure (48 hr, 50 ng/ml, single dose). Shades of red and blue indicate relative levels of up- or down-regulation respectively. The Heatmap was generated with Morpheus (https://software.broadinstitute.org/morpheus).

To examine changes in protein expression we conducted proteomic analyses on the cells following Acute (48 hour) and Chronic (7 day) exposure with Prague PM (50 ng/ml) and used bioinformatic analyses to examine the most significantly affected pathways and processes. In total, 29 proteins were differentially expressed after acute exposure. These are listed in Fig 3 with their fold change compared to unexposed control cells, and replicate values represented in a heat map, where red and blue indicate up-and down-regulation, respectively. Of the 29 differentially expressed proteins, three were down-regulated and 26 were up-regulated (Fig 3).

**Fig 4:**
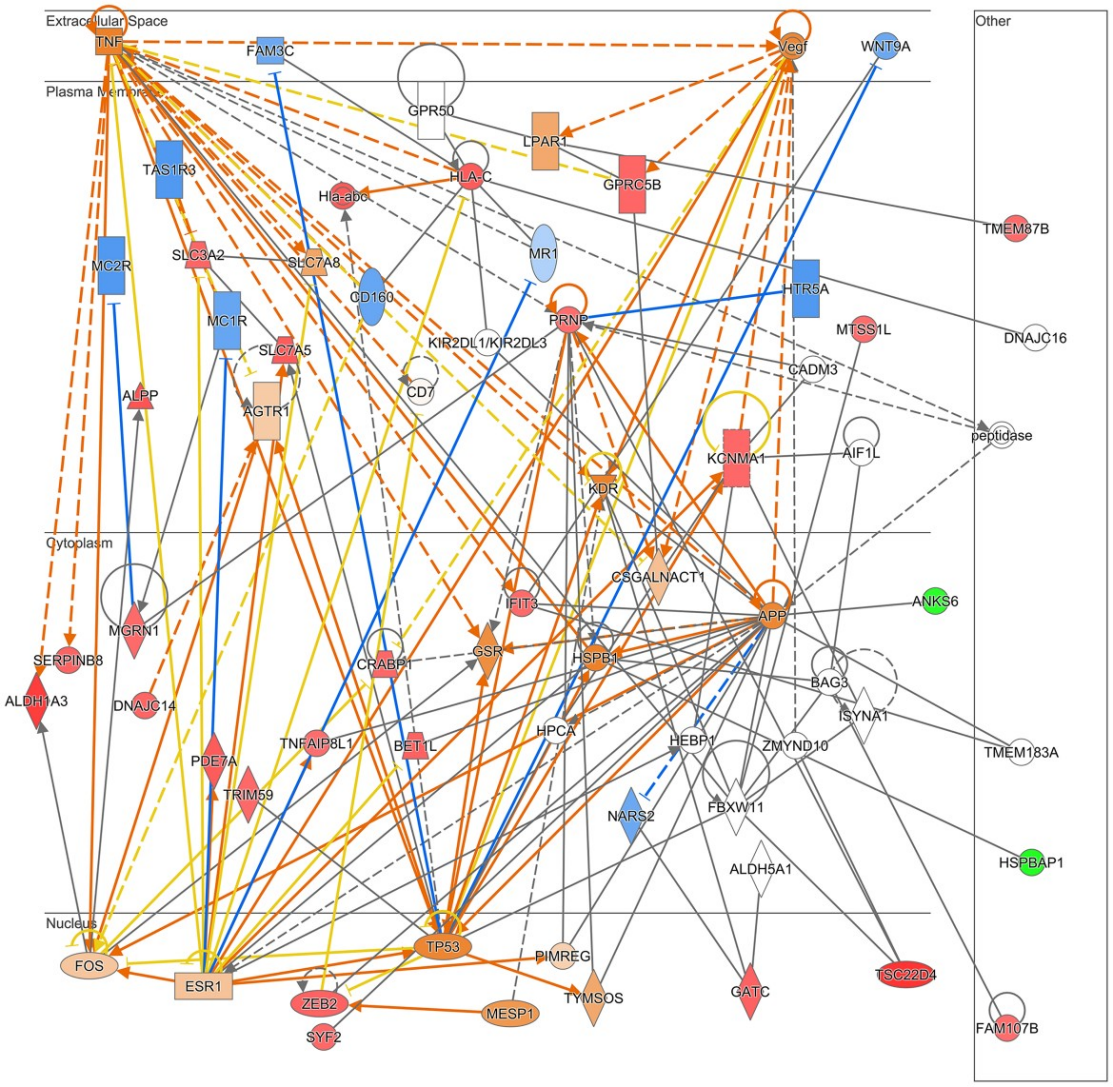
Top networks affected in trophoblast cells acute Prague PM exposure. IPA network diagram showing combination of the top two networks (‘Amino acid metabolism, molecular transport, small molecule biochemistry’ and ‘Cell death and survival, cellular compromise, cancer’) affected in trophoblast cells exposed to Prague PM (50 ng/ml) for 48 h. Actual up- and down-regulated proteins are shown in red and green respectively, predicted activated and inhibited proteins are shown in orange and blue respectively, orange lines indicate activation, blue lines inhibition, yellow lines indicate inconsistent findings and grey lines no effect predicted. The Networks & Functional analyses were generated through the use of Ingenuity Pathways Analysis, Qiagen.

The IPA analyses of acute PM exposure indicated that the most significant pathways affected were phagosome maturation, retinoic acid receptor (RAR) activation (p<0.02). However, of the proteins associated with these canonical pathways only 2/148 and 2/190 proteins, respectively showed expression changes. Clustering of cellular functions into networks revealed 2 major networks (amino acid metabolism/molecular transports/small molecule biochemistry, and cell death and survival/cellular compromise/cancer). Merging these two networks revealed major nodes involving TNF and VEGF signalling in the extracellular space, amyloid beta precursor protein, prion protein in the membrane and TP53, Fos a zinc finger E-box binding homeobox factor (ZEB2) and the estrogen receptor in the nucleus (Fig 4).

**Fig 5:**
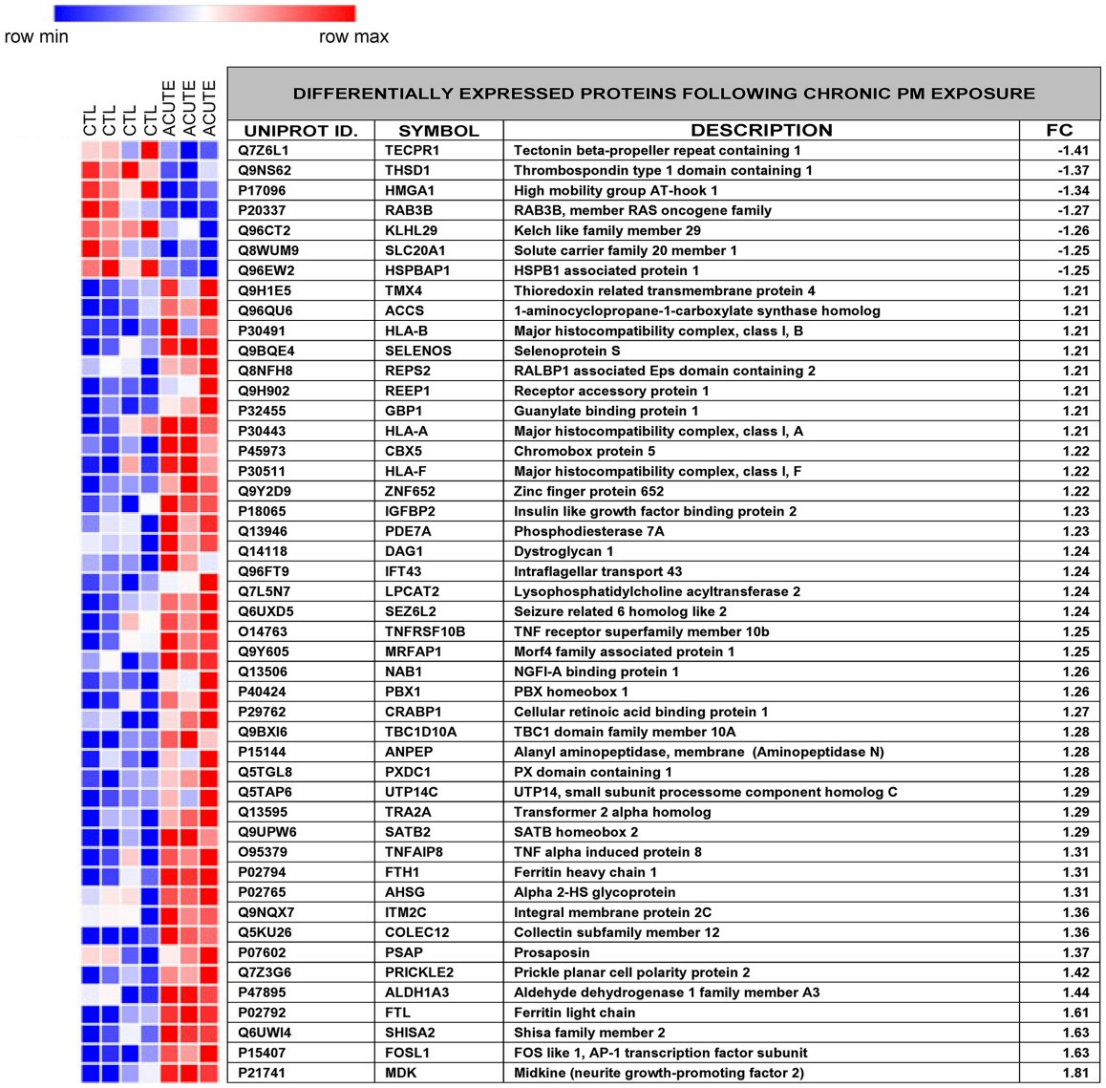
Differentially expressed proteins following chronic Prague PM exposure. Heatmap showing replicate expression intensities for control (CTL) and chronic exposed samples with matching table showing levels of differential protein (UniProt code and description) expression (FC, fold change) following chronic PM (7 day, 50 ng/ml daily) exposure. Shades of red and blue indicate relative levels of up- or down-regulation respectively. The Heatmap was generated with Morpheus (https://software.broadinstitute.org/morpheus).

Following chronic exposure to Prague PM (7 days, 50 ng/ml daily), 47 proteins were differentially expressed compared to unexposed controls (Fig 5). Of the 47 proteins, 7 were down regulated and 40 were upregulated.

**Fig 6:**
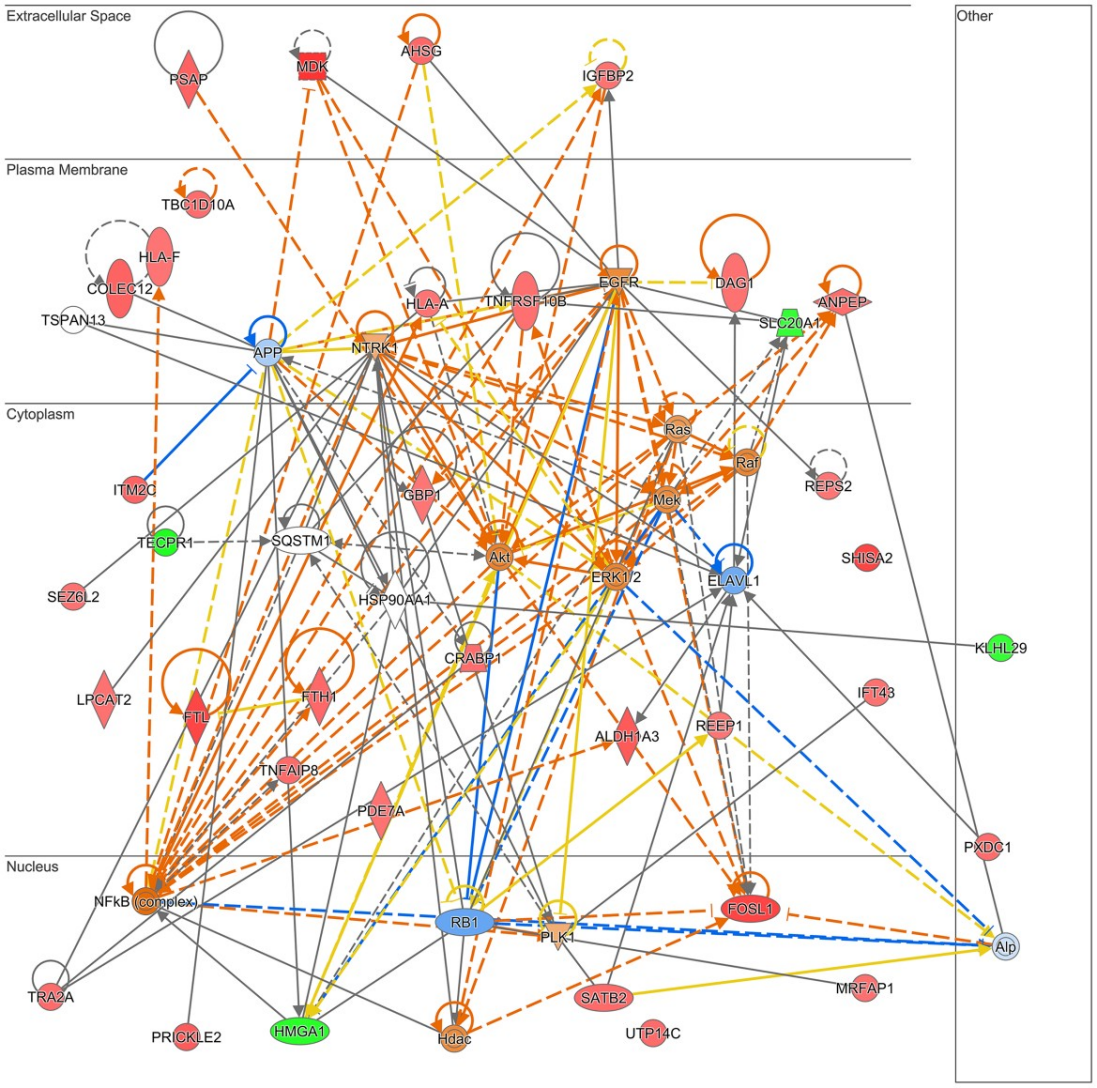
Top networks affected in trophoblast cells chronic Prague PM exposure. IPA network diagram showing combination of the top two networks (‘Cellular development, cellular growth and proliferation, connective tissue development and function’ and ‘Cell-to-cell signalling and interaction, drug metabolism, molecular transport’) affected in trophoblast cells following chronic Prague PM (7 days, 50 ng/ml daily) exposure. Actual up- and down-regulated proteins are shown in red and green respectively, predicted activated and inhibited proteins are shown in orange and blue respectively, orange lines indicate activation, blue lines inhibition, yellow lines indicate inconsistent findings, and grey lines no effect predicted. The Networks & Functional analyses were generated through the use of Ingenuity Pathways Analysis, Qiagen.

The top five canonical pathways affected were antigen presentation (p<0.0027; 2/38 proteins), autoimmune thyroid disease signalling (p<0.004; 2/47), graft-versus-host disease signalling (p<0.0042; 2/48), acute phase response signalling (p<0.0048; 3/170) and NRF2-mediated oxidative stress response (p<0.0069; 3/193). Clustering of cellular functions into networks revealed 2 major networks (cellular movement/cell cycle/cellular development and cell death and survival/cancer/organismal injury and abnormalities). Merging of these networks implicated 36 focus molecules involved in extracellular signalling (VEGF, Midkine, IGFBP2, immunoglobulins, interferon alpha) acting through membrane proteins (amyloid precursor protein, TNFRSF10B, NTRK1 EGFR), converging on the Ras-MAPK cascade and AKT in the cytoplasm and Fos/Jun, NFkB, RB1 and HMGA1 in the nucleus (Fig 6).

We initially used western blotting analysis to validate proteomic findings but given the low percentage changes in protein abundance, this method was not sensitive enough to show the differences between control and PM exposed cells (see S3A Fig). Instead we used RPPA analysis, which confirmed the pathway activation predicted by IPA (see S3B Fig). Notably, TP53 following acute PM exposure, as well as AKT and ERK, following chronic PM exposure, were activated. These proteins are highlighted in orange in Figs 4 and 6, respectively.

## Discussion

There is a strong association between levels of PM pollution and pregnancy complications such as preterm births and pre-eclampsia ([3–7], disorders in which placental dysfunction plays a pivotal role [16]. Inhaled particles can be detected in the circulation within 1 minute [27] and a recent study has shown that particulate matter can be found within cells in the placenta [34], suggesting this tissue is also a target for air pollution.

Similarly, rodents exposed to PM via inhalation during gestation developed morphological changes in the placenta [47]. Moreover, inhaled particles, such as nanoparticles, can reach the mouse placenta [26], and exposure to diesel particles can cause DNA damage [48] and increased cytokine production in the fetus [25] and in the placenta [49], suggesting transplacental transfer of pollution particles from the maternal to the fetal circulation. Consistent with this, analysis of samples of full-term placenta exposed to air pollution PM showed high levels of heavy metals in the syncytiotrophoblast layers and it was proposed that trophoblast cells actively phagocytosed and concentrated metal-containing particles [50].

We attempted in this study to calculate a realistic exposure rate based on our previous epidemiological data studies showing that there is an association between women exposed to ambient air pollution in Malmö and low birth weights and pre-term births [3–4]. As the average daily mean ambient PM2.5 is between 20–30 μg/m^3^ in Malmö, Sweden, (compared to Swedish national average < 10 μg/m^3^), we began our calculations assuming pregnant women were exposed to PM2.5 of 25 μg/m^3^ daily. We calculated that pregnant women were exposed to between 50–500 ng of PM2.5 per day. However, in other cities, such Taiyuan City, China [8] that have higher levels of average daily ambient levels of PM2.5, testing higher concentrations in the microgram to low milligram range on trophoblast cells would be highly appropriate.

In this study we have shown, using an *in vitro* approach that PM particles are internalised by endocytosis in human trophoblast cells for at least 7 days, with many of the internalised vesicles accumulating in the perinuclear region. Moreover, the initiation of particle endocytosis is very rapid, occurring within 30 minutes of exposure with both Malmö and Prague PM. While at this time-point there was no clear effect on growth of the cells, the number of cells in cultures exposed to PM for 7 days was significantly decreased, suggesting PM exposure inhibits cell growth in vitro. In addition to changes in growth, there are alterations in a trophoblast pregnancy marker (hCGβ) and inflammation (IL-6) but not in placental differentiation (progesterone) within 48 hours. By contrast, Wang and colleagues [51] reported a significant decrease in progesterone levels following exposure of trophoblast cells to industry-derived PM2.5. While we do not know the reason for the differences in results between studies, we have used HTR-8/SVneo cells (a first trimester transformed cell line from human chorionic villi explants), and Wang et al used JEG-3 cells, which are derived from a human choriocarcinoma. Thus, it is possible the difference in results relate to the different origin of the cell lines used.

While by RT-PCR there were no significant changes in several genes that have been associated with hypoxia (*NFE2L2*, *HIF1A*, *COX10*, *HMOX1*) and cell adhesion (*MMP9*, *PECAM1* and *ITGA5*) after 48 hours exposure, the proteomic analyses of cells exposed to Prague PM, acutely (48 h) or chronically (7 days), showed significant changes in 29 and 47 proteins respectively. These findings confirm that PM enter trophoblast cells by endocytosis and can alter cellular pathways.

The acute inflammatory (IL-6) and dysfunctional (hCGβ) responses observed in the HTR-8/SVneo cells are consistent with earlier studies, which showed an increase in the release of proinflammatory cytokines (e.g. IL-6, IL-1b) into the human circulation following exposure to PM [17]. Further support for an inflammatory response is provided by increases in interferon-induced protein (IFIT3, [52]), and a phosphodiesterase (PDE7A, [53]), which have been associated with innate immune system activation and inflammation, respectively, and are upregulated following acute PM exposure. Both Acute and Chronic PM exposure led to increases in PDE7A, which has been suggested as a target to alleviate inflammation associated with various inflammatory diseases [53, 54] and underscores the activation of inflammation in trophoblast cells following exposure to air pollution.

The acutely increased expression of prion protein (PRNP, [55]), chaperone proteins, DNAJC14 [56] and FAM107B [57], the endo-lysosomal trafficking protein (MGRN1) [58] and aldehyde dehydrogenase (ALDH1A3) [59], which can be upregulated following acute cell stressors, suggests that the PM-treated trophoblast cells exhibit a stress response. The up-regulation of TP53, suggests that these cells may be more prone to cell death. Consistent with this, there was an observed loss of cells from these cultures with time. Changes in human placental functions including altered gene expression and increases in markers of oxidative stress [28, 29], mitochondrial dysfunction and altered methylation profiles [30–32], and endothelial and villi pathology [21] have been reported following inhalation of PM during pregnancy.

While the Acute response to PM appeared to affect amino acid metabolism, molecular transport and cell death and survival, the predominant chronic response to PM appeared to involve changes in immune signaling affecting antigen presentation, autoimmune responses and oxidative stress responses. While neither the Prague nor Malmö PM have been analysed for bioaerosols, other traffic-pollution particulate matter, including PM2.5 and PM10, have been shown to contain endotoxins, which cause inflammatory and innate immune responses in the respiratory system [60–62]. Consistent with this, the clustering of the dysregulated proteins into the ontologies of antigen presentation, acute phase response signalling, NRF2-mediated oxidative stress response and innate immune system activation, following chronic PM exposure suggest that the Malmö and Prague particles may contain microorganism fragments or endotoxins that activate inflammatory and innate immune responses in placental cells. Bacterial endotoxins can trigger inflammation via interactions with Toll-like receptors (TLR/CD14) [63] and the changes in IFIT3 and IL-6 at 48 h as well as the implication of NFkB involvement in the chronically exposed cells at 7 days are consistent with an endotoxin response [52].

The upregulated expression of endocytosis and intracellular transport proteins (REPS2, IFT43, TBC1D10A, PXDC1, RAB35,) are consistent with the uptake of PM into endosomes or phagosomes, which normally would eventually fuse with lysosomes for degradation. The accumulation of vesicular PM in the perinuclear region for up to 7 days suggests that the process of degradation is dysfunctional. Consistent with this, there is dysregulated expression of KLH29, RAB35 and TCPR1 proteins, which are involved in fusing endosomes to lysosomes. Moreover, the expression of ER stress related proteins TMX4 [64], REEP1 [65], TNFAIP8 [66]) and cell death associated proteins (TNFRSF10B [67], TRAIL [68], suggest that the PM has disrupted ER-phagosome interactions [69] and induced an ER stress-like response.

Various stimuli can lead to ER stress, including endotoxins, drugs and inflammation followed by overloaded or misfolded proteins [70]. Therefore, alternative mechanisms by which PM exposure may lead to ER stress in trophoblast cells is by increased internalisation of membrane receptors, and innate immune system activation, via the upregulation of AMPN (also known as CD13) and GBP1, which are known to regulate Toll-Like receptor internalisation (63) or antigen internalisation respectively (64). This may lead to increased activation of signalling cascades, increased inflammation as evidenced by LPCAT2 and MDK, which are known to be upregulated during inflammation (65, 66) and dysregulation of chaperone proteins (increases in DNAJC1, FAM107B and decrease in HSPBAP1) as shown in this study. Importantly, trophoblast cells are known to induce platelet-activating factor (PAF) upon inflammatory stimulation, via the activity of acetyl-transferase, LPCAT2, which was upregulated (+1.24 fold) in the chronically exposed cells. The fact that acetylated-PAF was found to be much higher in PE than in normal placentas (67), reinforces the parallels between PM-exposed trophoblasts and dysfunctional placentas in PE.

An intriguing finding in the acute response was the dysregulated expression of several metal ion transport proteins (SLC3A2, SLC7A5, TRIM59, KCNMA1). As both Malmö PM2.5 (See Supplementary Information S1 Table) and Prague PM10 contain metals including mercury, copper, iron, zinc and lead, it is plausible that these expression changes are adaptive responses by the cell to cope with these heavy metals and thus modulate oxidative stress that can lead to superoxide and hydroxyl radicals [63]. In this context the expression of PRNP protein may provide a link between metal exposure and cell signalling as it has been implicated in diverse cellular functions including placenta development, metal ion transporter, signalling and cytoskeletal associated protein [71]. In the IPA network analyses for Acute exposure, PRNP upregulation was linked to activation of several signalling proteins including ERK1, P38 MAPK, FOSL1 and TP53, as well as chaperone proteins (HSPB1, DNAJC14) and APP. Similar to the compensatory expression of ion transport genes, the dramatic increases (44–66%) in ALDH1A3 following chronic or acute PM exposure may be a response to the high levels of various aldehydes that are present in urban traffic-derived PM. AKT and ERK pathways were predicted to be activated by IPA analysis and these were confirmed to show increased phosphorylation by the RPPA analyses.

The differentially expressed proteins detected in the PM-exposed HTR-8/SVneo cells are similar to proteins and genes that are differentially expressed in PE and IUGR. For instance, PRNP is highly upregulated in PE compared to normal pregnancy levels [72] and transcriptome profiling of 200 placentas, revealing that DNAJC14 was one of five hub proteins that were significantly associated with fetal growth restriction [73]. Other differentially expressed proteins found in our study, following Acute (FAM107B) and Chronic PM exposure (PRICKLE2, TNFAIP8, DAG1, PSAP, IFT43 and ZNF652) were also present in placental expression networks associated with birth weight restriction [73]. This leads to the suggestion that PM exposed trophoblast cells may activate similar cell stress pathways as occurs in PE and foetal growth restriction.

## Conclusions

It is now well-established that different sized inhaled PM can cross into the human circulation directly, or indirectly via phagocytosis by immune cells that migrate to the lymphatic system where most are cleared via the kidney and gastrointestinal tract [39, 74, 75], and can reach the placenta [34]. Our studies suggest that even low levels of urban PM can have damaging effects on trophoblast cells, even over short exposure periods. Trophoblast cells actively take up urban PM by endocytosis and may respond to the particles themselves, or their components such as endotoxins, reactive metals, or PAHs. The accumulation of the vesicular-bound particles in the perinuclear region, appears to lead to changes in trafficking proteins with subsequent activation of ER stress, growth inhibition, oxidative stress and inflammation. Many of the dysfunctional cellular processes ascribed to the differentially expressed proteins in this study are similar to those found in PE and IGUR, suggesting that air pollution particulate matter may contribute to these conditions.

## Supporting information

S1 Fig Effect of PM exposure on trophoblast progesterone secretion. The level of Progesterone secretion was measured in culture supernatant following exposure for 48 hours with varying doses of Prague (**A**) or Malmö (**B)** PM or PM-conditioned media. Results are expressed as ± S.D of triplicate wells. * p < 0.05. Abbreviations: Control (**CTRL**), PM-conditioned media (**S-**).
(TIF)

S2 Fig Stained trophoblast cells used to prepare animation for S1 Movie. This fig shows corresponding stained cells (Nucleus-DAPI Blue staining, and vesicles-PKH67 green staining) used to prepare z-stack and its animation.
(TIF)

S3 Fig Validation of proteomic studies. S3 Fig A shows the results of western blotting probing for the upregulated proteins following chronic exposure to PM: ALPP, TSCD22D4, PDE7A and downregulated HSPBAP1. The very small changes in protein abundance identified via proteomics and SRM, were difficult to detect using this methodology. S3 Fig B shows the results of RPPA analysis following chronic and acute exposure to PM. These results confirm the predictions of activated pathways following IPA analysis, namely AKT and ERK following chronic PM exposure, and TP53 following acute PM exposure.
(JPG)

S1 Movie Animation showing internalised Prague PM in trophoblast cells. *Prague PM10 24 hr Exposure*.*avi*; Z-stack animation, showing the presence of internalised vesicles with PKH7 staining in cells exposed to PM after 24 hr exposure.
(AVI)

S1 Table Concentration of different PAHs and metals from urban PM2.5 samples collected in Malmö, Sweden. Table showing the concentration (ng/mg) of 32 individual PAHs and 14 metals determined in the urban PM2.5 samples collected in Malmö, Sweden. The PAH levels found in the Malmö PM sample were somewhat lower, a factor of 1.5–5 dependent on compound, compared to published results of the PM Prague sample (NIST Certificate of Analysis Standard Reference Material 2786, 2016, Gaithersburg, MD, USA). Notably, even greater differences were found for the metals, a factor 2 to 100 times higher for the individual metals, in the Prague PM samples than in the Malmö PM sample. Analysis of nitro-PAHs and oxy-PAHs were not included in this study but will be the focus of future work.
(DOCX)

S2 Table Effect of PM exposure on trophoblast gene expression. The expression of selected genes (GAPDH, 18S, NFE2L2, MMP9, COX10, HMOX1, HIF1A, PECAM1 and ITGA5) in trophoblast cells following exposure of varying doses (ng/ml) of Prague (S4 Table A) or Malmö (S4 Table B) PM for 48 hours. Values are based on relative expression (ΔΔCt values, using methodology developed by Pfaffl, 2001^1^) compared to unexposed control cells. Values in the Table are Average (av) ± Standard Deviation (std), n = 6 per sample per gene per PM concentration, compared to unexposed control cells equal to 1. We found no significant changes in expression for any gene at any exposure concentration. ^1^Pfaffl, M. W (2001). A new mathematical model for relative quantification in real-time RT-PCR. Nucleic Acids Research. 29:2002–2007.
(DOCX)

S1 Text Physiological dose considerations, methodology and rationale for gene expression analysis. a. Physiological dose considerations based on Malmo average daily ambient concentration of PM2.5. b. Methodology and rationale for gene expression analyses of PM exposed trophoblast.
(PDF)

S2 Text Copyright Permission. Declaration assigning copyright for Figs 4 and 6, which were generated under licence, using Ingenuity Pathway Analysis Software (Qiagen), by author Robbert de Iongh.
(PDF)

## Abbreviations

DE: differentially expressed
ER: endoplasmic reticulum
PAHs: polyaromatic hydrocarbons
PE: pre-eclampsia
PM: particulate matter
