# Supplementary figures and images for: Exposure of trophoblast cells to fine particulate matter air pollution leads to growth inhibition, inflammation and ER stress
Source: PLoS One. 2019 Jul 18;14(7):e0218799. doi: 10.1371/journal.pone.0218799 (PMC6638881; doi:10.1371/journal.pone.0218799)

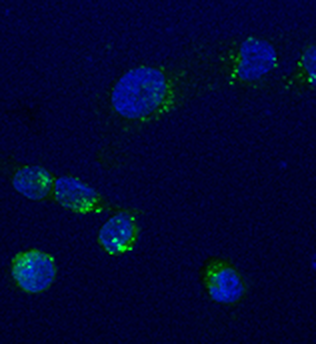

Supplement: S1 Fig — The level of Progesterone secretion was measured in culture supernatant following exposure for 48 hours with varying doses of Prague (A) or Malmö (B) PM or PM-conditioned media. Results are expressed as ± S.D of triplicate wells. * p < 0.05. Abbreviations: Control (CTRL), PM-conditioned media (S-). (TIF) [file pone.0218799.s001.TIF]

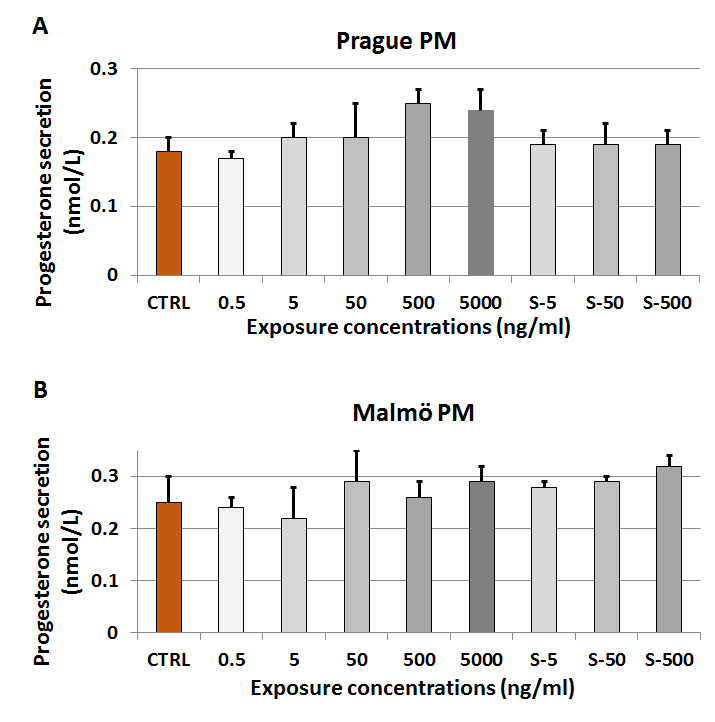

Supplement: S2 Fig — This fig shows corresponding stained cells (Nucleus-DAPI Blue staining, and vesicles-PKH67 green staining) used to prepare z-stack and its animation. (TIF) [file pone.0218799.s002.tif]

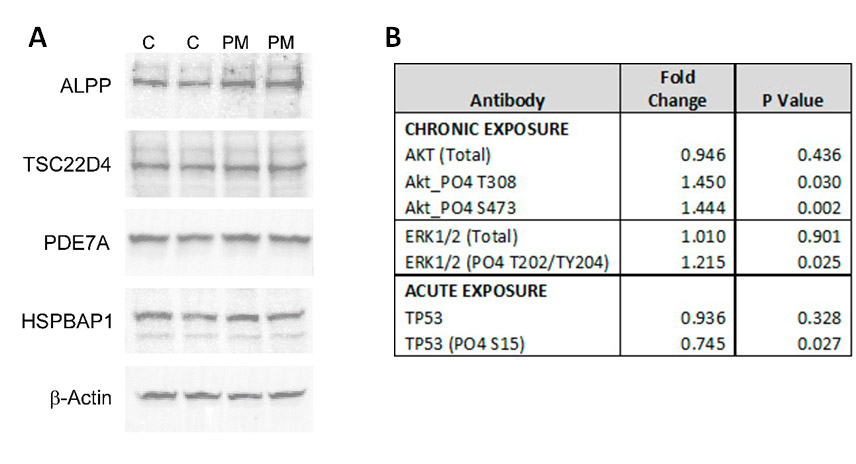

Supplement: S3 Fig — A shows the results of western blotting probing for the upregulated proteins following chronic exposure to PM: ALPP, TSCD22D4, PDE7A and downregulated HSPBAP1. The very small changes in protein abundance identified via proteomics and SRM, were difficult to detect using this methodology. S3 Fig B shows the results of RPPA analysis following chronic and acute exposure to PM. These results confirm the predictions of activated pathways following IPA analysis, namely AKT and ERK following chronic PM exposure, and TP53 following acute PM exposure. (JPG) [file pone.0218799.s003.jpg]
