# Supplementary material for: Exposure of trophoblast cells to fine particulate matter air pollution leads to growth inhibition, inflammation and ER stress
Source: PLoS One. 2019 Jul 18;14(7):e0218799. doi: 10.1371/journal.pone.0218799 (PMC6638881; doi:10.1371/journal.pone.0218799)
Supplement: S1 Table — Table showing the concentration (ng/mg) of 32 individual PAHs and 14 metals determined in the urban PM2.5 samples collected in Malmö, Sweden. The PAH levels found in the Malmö PM sample were somewhat lower, a factor of 1.5–5 dependent on compound, compared to published results of the PM Prague sample (NIST Certificate of Analysis Standard Reference Material 2786, 2016, Gaithersburg, MD, USA). Notably, even greater differences were found for the metals, a factor 2 to 100 times higher for the individual metals, in the Prague PM samples than in the Malmö PM sample. Analysis of nitro-PAHs and oxy-PAHs were not included in this study but will be the focus of future work. (DOCX) [file pone.0218799.s005.docx]

| **PAH** | **Concentration (ng/mg)** | **Metal** | **Concentration (ng/mg)** |
| --- | --- | --- | --- |
| *benzo(b)fluoranthene* | 4.2 | Iron (Fe) | 890 |
| *chrysene* | 3.9 | Aluminium (Al) | 330 |
| *benzo(k)fluoranthene* | 3.1 | Zink (Zn) | 240 |
| *pyrene* | 3.0 | Copper (Cu) | 100 |
| *fluoranthene* | 2.7 | Vanadium (V) | 36 |
| *benzo(g,h,i)perylene* | 2.6 | Manganese (Mn) | 34 |
| *indeno(1,2,3-c,d)pyrene* | 2.1 | Barium (Ba) | 17 |
| *benzo(a)anthracene* | 1.3 | Lead (Pb) | 14 |
| *phenanthrene* | 0.96 | Nickel (Ni) | 14 |
| *benzo(a)pyrene* | 0.77 | Arsenic (As) | 5.3 |
| retene | 0.60 | Chromium (Cr) | 4.8 |
| *dibenzo(a,h)anthracene* | 0.39 | Cobalt (Co) | 1.8 |
| 2-methylphenanthrene | 0.34 | Cadmium (Cd) | 0.49 |
| 2-methylchrysene | 0.31 | Thallium (Tl) | 0.061 |
| 2-phenhylnaphtalene | 0.29 |  |  |
| 3-methylphenanthrene | 0.26 |  |  |
| 1-methylphenanthrene | 0.25 |  |  |
| 1-methylanthracene | 0.24 |  |  |
| 1-methylpyrene | 0.17 |  |  |
| perylene | 0.15 |  |  |
| *fluorene* | 0.075 |  |  |
| *anthracene* | 0.075 |  |  |
| 2-methylnaphtalene | <0.052 |  |  |
| *acenaphtnene* | <0.051 |  |  |
| *acenaphthylene* | 0.044 |  |  |
| *naphtalene* | <0.039 |  |  |
| 1-methylfluorene | 0.038 |  |  |
| 1-methylnaphtalene | <0.031 |  |  |
| 2,3-dimethylnaphtalene | <0.012 |  |  |
| biphenyl | <0.0082 |  |  |
| 2,3,5-trimethylnaphtalene | <0.0062 |  |  |
| 1-methylfluoranthene | <0.00034 |  |  |
|  |  |  |  |
| Sum PAHs | 28 |  |  |
| Sum 16 US EPA PAHs | 25 |  |  |
| Sum acrylated PAHs | 2.6 |  |  |

**S1 Table.** **Concentration of 32 PAHs, the 16 US EPA PAHs and 16 alkylated PAHs, and metals (ng/mg) from urban PM2.5 samples collected in Malmö, Sweden.**

Table showing the concentration (ng/mg) of 32 individual PAHs and 14 metals determined in the urban PM2.5 samples collected in Malmö, Sweden**.** The PAH levels found in the Malmö PM sample were somewhat lower, a factor of 1.5-5 dependent on compound, compared to published results of the PM Prague sample (NIST Certificate of Analysis Standard Reference Material 2786, 2016, Gaithersburg, MD, USA). Notably, even greater differences were found for the metals, a factor 2 to 100 times higher for the individual metals, in the Prague PM samples than in the Malmö PM sample. Analysis of nitro-PAHs and oxy-PAHs were not included in this study but will be the focus of future work.
