# Supplementary material for: Exposure of trophoblast cells to fine particulate matter air pollution leads to growth inhibition, inflammation and ER stress
Source: PLoS One. 2019 Jul 18;14(7):e0218799. doi: 10.1371/journal.pone.0218799 (PMC6638881; doi:10.1371/journal.pone.0218799)
Supplement: S2 Table — The expression of selected genes (GAPDH, 18S, NFE2L2, MMP9, COX10, HMOX1, HIF1A, PECAM1 and ITGA5) in trophoblast cells following exposure of varying doses (ng/ml) of Prague (S4 Table A) or Malmö (S4 Table B) PM for 48 hours. Values are based on relative expression (ΔΔCt values, using methodology developed by Pfaffl, 20011) compared to unexposed control cells. Values in the Table are Average (av) ± Standard Deviation (std), n = 6 per sample per gene per PM concentration, compared to unexposed control cells equal to 1. We found no significant changes in expression for any gene at any exposure concentration. 1Pfaffl, M. W (2001). A new mathematical model for relative quantification in real-time RT-PCR. Nucleic Acids Research. 29:2002–2007. (DOCX) [file pone.0218799.s006.docx]

**S2 Table**

The expression of selected genes (GAPDH, 18S, NFE2L2, MMP9, COX10, HMOX1, HIF1A, PECAM1 and ITGA5) in trophoblast cells following exposure of varying doses (ng/ml) of Prague (S4 Table A) or Malmö (S4 Table B) PM for 48 hours. Values are based on relative expression (ΔΔCt values, using methodology developed by Pfaffl, 2001^1^) to unexposed control cells. Values in the Table are Average (av) ± Standard Deviation (std), n=6 per sample per gene per PM concentration, compared to unexposed control cells equal to 1. We found no significant changes in expression for any gene at any exposure concentration.

| **S4 Table A. Prague PM** | | | | | | | | | | | | | | |
| --- | --- | --- | --- | --- | --- | --- | --- | --- | --- | --- | --- | --- | --- | --- |
|  | **NFE2L2** | | **MMP9** | | **COX10** | | **HMOX1** | | **HIF1A** | | **PECAM1** | | **ITGA5** | |
| **ng/ul** | **av** | **std** | **av** | **std** | **av** | **std** | **av** | **std** | **av** | **std** | **av** | **std** | **av** | **std** |
| **0.5** | 1.71 | 1.07 | 0.84 | 0.24 | 1.05 | 0.65 | 0.98 | 0.54 | 1.36 | 0.59 | 1.55 | 0.73 | 1.12 | 0.55 |
| **5** | 1.04 | 0.61 | 0.4 | 0.3 | 0.24 | 0.1 | 0.23 | 0.13 | 1.05 | 0.47 | 1.36 | 0.35 | 0.32 | 0.16 |
| **50** | 1.3 | 0.45 | 0.6 | 0.22 | 0.75 | 0.38 | 0.57 | 0.26 | 1.29 | 0.41 | 1.43 | 0.3 | 0.46 | 0.16 |
| **500** | 0.68 | 0.3 | 0.53 | 0.21 | 0.73 | 0.31 | 0.72 | 0.3 | 0.56 | 0.27 | 0.64 | 0.31 | 0.48 | 0.24 |
| **5000** | 1.21 | 0.48 | 0.58 | 0.27 | 0.73 | 0.21 | 0.73 | 0.4 | 1.09 | 0.43 | 1.04 | 0.67 | 0.81 | 0.53 |

| **S4 Table B. Malmö PM** | | | | | | | | | | | | | | |
| --- | --- | --- | --- | --- | --- | --- | --- | --- | --- | --- | --- | --- | --- | --- |
|  | **NFE2L2** | | **MMP9** | | **COX10** | | **HMOX1** | | **HIF1A** | | **PECAM1** | | **ITGA5** | |
| **ng/ul** | **av** | **std** | **av** | **std** | **av** | **std** | **av** | **std** | **av** | **std** | **av** | **std** | **av** | **std** |
| **0.5** | 1.16 | 0.26 | 0.91 | 0.16 | 0.93 | 0.15 | 0.89 | 0.13 | 1 | 0.12 | 1.11 | 0.14 | 0.78 | 0.19 |
| **5** | 1.39 | 0.49 | 0.82 | 0.1 | 0.81 | 0.06 | 0.76 | 0.14 | 0.71 | 0.12 | 0.79 | 0.22 | 0.97 | 1.19 |
| **50** | 1.18 | 0.21 | 0.96 | 0.17 | 0.9 | 0.15 | 0.8 | 0.12 | 0.88 | 0.15 | 1.1 | 0.3 | 0.98 | 0.28 |
| **500** | 1.03 | 0.14 | 0.75 | 0.24 | 1.13 | 0.18 | 0.58 | 0.12 | 1.44 | 0.52 | 1.1 | 0.64 | 0.86 | 0.52 |
| **5000** | 1.22 | 0.28 | 1.21 | 0.15 | 1.03 | 0.15 | 0.87 | 0.24 | 1.61 | 0.57 | 0.7 | 0.19 | 1.08 | 0.27 |

^1^Pfaffl, M. W (2001). A new mathematical model for relative quantification in real-time RT-PCR. Nucleic Acids Research. 29:2002-2007.
