# Supplementary material for: Exposure of trophoblast cells to fine particulate matter air pollution leads to growth inhibition, inflammation and ER stress
Source: PLoS One. 2019 Jul 18;14(7):e0218799. doi: 10.1371/journal.pone.0218799 (PMC6638881; doi:10.1371/journal.pone.0218799)
Supplement: S1 Text — a. Physiological dose considerations based on Malmo average daily ambient concentration of PM2.5. b. Methodology and rationale for gene expression analyses of PM exposed trophoblast. (PDF) [file pone.0218799.s007.pdf]

S1 File

a. Physiological dose considerations based on Malmo average daily ambient PM2.5

The actual exposure rate of the placenta to PM is not known, although it is well established that PM ≤ 2.5 µm, can and do bypass lung epithelial phagocytic cells to enter directly into the circulation (38). In order to estimate a realistic exposure dose that the placenta receives, the following criteria were considered.

1. the average daily mean ambient PM2.5 of 20 - 30 µg/m^3 in Malmö Sweden (http://aqicn.org/city/sweden/malmo-radhuset/) and Prague, Czech Republic (25 - 70 µg/m^3, (http://aqicn.org/city/czechrepublic/prague/pha4-libus/). We assumed women were exposed to an average ambient PM2.5 of 25 µg/m^3 daily for our calculations.
2. changes in cardiorespiratory physiology during pregnancy (29).
3. the abundance of leukocytes particularly macrophages in the different compartments of the adult lung (30) and assuming a clearance capacity of 90% where it is cleared via the gastrointestinal tract or lymph nodes, and that excess PM up to 4 µm can move into the circulation, and up to 10% PM4 and 50% PM0.1 are deposited into the pulmonary alveolar region (28).
4. the assumption that pregnant women are exposed to PM only when outdoors (approximately 3 hours per day) and breathing via the mouth at a normal rate (that is, not exercising);

Based on these considerations, we have estimated the placenta is exposed to approximately 50-500 ng PM per day used these as the daily exposure rate for cultured trophoblast cells. While acknowledging that *in vitro* exposure does not reflect *in vivo* deposition in the lungs and circulation, the biological effects of PM on the placenta cannot be ascertained in pregnant women. We have used the concentration of PM in air as the initial exposure metric (µg/m^3) rather than inhaled dose (mg/kg-day; Integrated Risk Information System [Internet]. USA, EPA. 2017. Available from: https://www.epa.gov/iris.).

28. Kastury F, Smith E, Juhasz AL. A critical review of approaches and limitations of inhalation bioavailability and bioaccessibility of metal(loid)s from ambient particulate matter or dust. Sci Total Environ. 2017;574:1054-74.
29. Soma-Pillay P, Nelson-Piercy C, Tolppanen H, Mebazaa A. Physiological changes in pregnancy. Cardiovascular journal of Africa. 2016;27(2):89-94.
30. Tschernig T, Pabst R. What is the clinical relevance of different lung compartments? BMC pulmonary medicine. 2009;9:39.

b. Methodology and rationale for gene expression analyses of PM exposed trophoblast.

To isolate RNA cells were washed twice, trypsinised, pelleted and resuspended in 300 µl RNA lysis buffer and processed using the RNeasy Mini Kit (Qiagen) according to manufacturer's instructions, then quantified using a Nanodrop (ThermoFisher).
Taqman custom arrays (Applied Biosystems) containing the following primers GAPDH, 18S, Nuclear Factor, Erythroid 2 Like 2 (NFE2L2[1]), Matrix Metallopeptidase 9 (MMP9[2]), Cytochrome C Oxidase Homolog 10 (COX10[3]), Heme Oxygenase 1 (HMOX1[1]), Hypoxia Inducible Factor 1 Alpha Subunit (HIF1A[4]), Platelet and Endothelial Cell Adhesion Molecule 1 (PECAM1[5]) and Integrin Subunit Alpha 5 (ITGA5[6]) were used to analyse gene expression following PM exposure. These genes have been reported to have altered gene expression in PE

placentas. Each dose of PM was analysed in triplicate wells, and each sample was analysed in duplicate for qPCR analysis. 100 ng total RNA was converted to first strand cDNA using Express One-step Superscript (Invitrogen) in 20 µl total volume according to manufacturer's instructions. 2µl template cDNA was combined with 10 µl Taqman Fast Advanced Master Mix (Applied Biosystems) according to manufacturer's instructions before qPCR analysis using Quant Studio 5 Real-Time PCR system (Applied Biosystems).

References:

[1] Li et al., (2016) Increased Heme Oxygenase-1 and Nuclear Factor Erythroid 2-Related Factor-2 in the Placenta Have a Cooperative Action on Preeclampsia. Gynecol Obstet Invest. 2016;81(6):543-551. Epub 2016 Oct 21.

[2] Laskowska (2017) Altered Maternal Serum Matrix Metalloproteinases MMP-2, MMP-3, MMP-9, and MMP-13 in Severe Early- and Late-Onset Preeclampsia. Biomed Res Int. 2017;2017:6432426. doi: 10.1155/2017/6432426. Epub 2017 Jul 17.

[3] He et al., (2004) Reduced amount of cytochrome c oxidase subunit I messenger RNA in placentas from pregnancies complicated by preeclampsia. Acta Obstet Gynecol Scand. 2004 Feb;83(2):144-8.

[4] Tal (2012) The role of hypoxia and hypoxia-inducible factor-1alpha in preeclampsia pathogenesis. Biol Reprod. 2012 Dec 13;87(6):134. doi: 10.1095/biolreprod.

[5] Goksu et al., (2012) Significance of platelet endothelial cell adhesion molecule-1 (PECAM-1) and intercellular adhesion molecule-1 (ICAM-1) expressions in preeclamptic placentae. Endocrine. 2012 Aug;42(1):125-31. doi: 10.1007/s12020-012-9644-9.

[6] Zhao et al (2017) FN1, FOS, and ITGA5 induce preeclampsia: Abnormal expression and methylation. Hypertens Pregnancy. 2017 Nov;36(4):302-309.
doi: 10.1080/10641955.2017.138579
